# Meeting Report: Pharmaceuticals in Water—An Interdisciplinary Approach to a Public Health Challenge

**DOI:** 10.1289/ehp.0901532

**Published:** 2010-03-25

**Authors:** Sara Rodriguez-Mozaz, Howard S. Weinberg

**Affiliations:** 1 Department of Environmental Sciences and Engineering, Gillings School of Global Public Health, University of North Carolina–Chapel Hill, Chapel Hill, North Carolina, USA; 2 Catalan Institute for Water Research, Parc Científic i Tecnològic de la Universitat de Girona, Girona, Spain

**Keywords:** drinking water, environment, natural waters, pharmaceuticals, risk assessment

## Abstract

**Background:**

The presence of pharmaceuticals in aquatic environments and in drinking water has prompted significant public interest regarding potential adverse ecological effects and risks to human health.

**Objectives:**

The Environmental Health Summit held in North Carolina, 10–11 November 2008, explored the issues associated with the presence and relative risk of trace levels of pharmaceuticals in water. More than 150 participants from government organizations and institutions, academia, industry, water utilities, and public interest groups participated in discussions aimed at evaluating the current knowledge on this issue and at identifying research gaps and innovative solution-oriented recommendations.

**Discussion:**

We present different aspects related to the subject that were discussed at the summit, including the source, fate, and transport of pharmaceuticals, their exposure effects and potential risks to human and ecosystems, and the best management practices to address these issues. Recommendations placed emphasis on research needs as well as education, communication, prevention, and intervention programs, and other public health solutions and actions.

**Conclusions:**

Despite rising concerns about the presence of trace amounts of pharmaceuticals in drinking water, little evidence is currently available that associates these chemicals with adverse human health risks. In order to prioritize which pharmaceutical chemicals could potentially pose the highest risk to consumers and the environment, the summit participants concluded that more studies are needed to generate meaningful and accurate data.

When drugs are prescribed prophylactically or in response to an acute or chronic illness, only a portion of the active ingredient of the drug is metabolized (Ternes 1998). The nonmetabolized parent compound, as well as the metabolites, enter the natural aquatic environment through waste discharges into receiving streams, which may reach downstream recreational lakes or even the intakes of drinking water treatment plants. The ubiquitous worldwide use of pharmaceuticals has led to the presence of low but measurable part-per-trillion levels of these biochemically active compounds in surface and groundwater. In the United States, the most comprehensive monitoring of these chemicals in water was performed between 1999 and 2000 by the U.S. Geological Survey in many of the nation’s streams (Kolpin et al. 2002). This study and others identified many products, including analgesics, antiinflammatories, antibiotics, antiepileptics, beta-blockers, blood lipid regulators, antidepressants, contrast media, oral contraceptives, and cytostatic and bronchodilator drugs in sewage, surface water, groundwater, and drinking water (Costanzo et al. 2005; Daughton and Ternes 1999; Glassmeyer et al. 2008; Heberer 2002; Stackelberg et al. 2007; Ternes 1998; Ye et al. 2007).

Pharmaceutical compounds are designed to have biochemical activity in target organisms at relatively low concentrations. Therefore, at the low part-per-trillion levels found in aquatic environments, which are far below human therapeutic doses, and even compared with the much higher levels of routinely measured water quality chemicals, there is concern that some of these compounds could have an ecological and human health effect. There is, for example, evidence from rodent and fish studies that suggests some endocrine-disrupting compounds, including those found in prescribed synthetic hormones, may contribute to tumor formation in humans (Birnbaum and Fenton 2003). The identification of pharmaceuticals in surface waters has opened a Pandora’s box in a way that other discoveries, such as the transmission of waterborne infectious diseases, have not. This might be because the subject brings to light that we may be drinking water that has, in part, been recycled through several other human bodies. Several countries have turned down opportunities to implement wastewater recycling to supplement potable water needs during drought conditions (Casani et al. 2005; Wilson and Pfaff 2008), but it is hard to conceal the fact that many people in those countries have been consuming wastewater-impacted drinking water for many years without any evidence of negative exposure-related health outcomes. Nevertheless, the presence of pharmaceuticals in surface waters and their detection, at much lower levels, in some household drinking waters has prompted significant public and mass media interest (Donn et al. 2008). The potential adverse ecological effects and the indirect human exposure to pharmaceutical chemicals warrant a multidisciplinary evaluation to establish a basis for appropriate risk assessment and potential environmental impact.

In the United States, the Food and Drug Administration (FDA) Center for Drug Evaluation and Research requires drug companies to submit environmental assessments for new drug applications (U.S. FDA 2009) with predicted wastewater treatment plant (WWTP) effluent introductory concentrations of > 1 ppb based on high-end projected sales and worse-case, end-of-pipe effluent discharges. However, the U.S. Environmental Protection Agency (EPA) is responsible for testing and regulating chemicals in drinking water, but pharmaceuticals are not currently regulated. In recognition of the growing concerns related to pharmaceuticals, one antibiotic and several hormones used in drug formulations are now on the Drinking Water Contaminant Candidate List (U.S. EPA 2010). Clearly, more information is needed to guide decisions about whether regulations on these chemicals are needed.

In light of these concerns, the Research Triangle Environmental Health Collaborative organized the Environmental Health Summit in the Research Triangle Park of North Carolina (USA) in November 2008 to identify the major issues surrounding public concern with the presence of pharmaceutically active chemicals in water. The collaborative was established to create a forum to promote partnerships and to set research priorities and public policy. This summit provided such a forum for more than 150 participants from North America and beyond, including stakeholders from the federal, state, and local governments, academic institutions, foundations, nonprofit organizations, drug manufacturing companies, and water utilities, as well as environmental and public health leaders. Thus, the goal of the summit was to provide a balanced consideration of this sensitive subject by recognizing its complexity and by involving multiple disciplines to assess all the related issues. During the meeting, a series of questions were tabled and a framework for a research agenda was created and built around the major issues that would address the balance of protecting public health through drug use while preventing unprescribed exposure from environmental input. In this article, we present an overview of the summit discussions.

## Issues

### Sources of pollution, fate, and transport

Scientific data regarding the source, fate, and transport as well as the environmental and human impact of veterinary and human pharmaceuticals would be beneficial to all stakeholders, including policy makers, to help assure that current use and disposal patterns offer no threat to society. However, such data as well as exposure assessment are limited and uncertain, in some cases generating challenges that can be considered in two major categories: source characterization and exposure assessment.

#### Source characterization

Pharmaceuticals are widely consumed by humans and also used in many animal feed commodities for treatment and prevention of disease and to enhance feed efficiency and growth. However, discrimination and apportionment between veterinary animal, agricultural, and human waste, whether from domestic or industrial sources, are not always clear, particularly because data on animal-waste pharmaceuticals are quite scarce. In the animal production industry, technologies have been developed to remove various waste products, including nutrients, heavy metals, ammonia, pathogenic bacteria, odors, and other parameters that are currently regulated by legislative mandates in some U.S. states. However, current wastewater treatment technologies, including those practiced at WWTPs, onsite processes, and even emerging animal waste treatments, do not specifically target pharmaceuticals for removal, so these chemicals are likely to be present at high levels in effluents from these processes (Cabello 2006; Dolliver and Gupta 2008; Kemper 2008). In fact, WWTPs are frequently identified as the main points of discharge of pharmaceuticals in surface waters (Radjenovic et al. 2007), but limited studies have been carried out to track the effectiveness of unit processes at these plants. Conducting such studies has become more challenging in part because plant managers are concerned that data showing the occurrence of pharmaceuticals in their waters could be misrepresented by the media (Donn et al. 2008). The use of stable chemical indicators of pollution for both veterinary and human pharmaceuticals was suggested as a relatively easy first step for tracking the fate and transport of pollutants from these sources without the need to identify every chemical present in the mix.

The quantity and quality of existing environmental occurrence data for pharmaceuticals are insufficient for decision making and may be questionable if the data are generated from nonstandardized analytical methods with insufficient quality assurance and control. For the most part, such data do not account for human or environmental metabolites or partitioning behavior between the aqueous and sediment phases in the natural environment. Furthermore, single-grab samples, which are the source of much of the data, do not adequately capture variation throughout the day, and, when used to measure environmental inputs, may not represent the true concentrations. Efforts should be made to choose more representative sampling methods that also take into account spatial variability (related to land and drug use patterns) and temporal variability and cycles (including longitudinal changes).

The ability to predict the fate and transport of chemicals in aquatic systems depends on the availability of physicochemical data that describe, among other characteristics, distribution coefficients, soil–water partitioning, and biodegradation rates. As part of the drug approval process in the United States, drug manufacturers must generate much of these data. If access to such data were improved, scientists would be in a better position to control and improve the design of water and WWTP technologies. The U.S. EPA is currently assembling a knowledge-based inventory that includes physical–chemical properties, occurrence data, analytical methods, WWTP influent and effluent measurements, and a bibliographic database for research.

#### Exposure assessment

Data on pharmaceutical degradation rates will assist in the design of models to predict their fate and characterization of processes that influence their transport, such as those in and between soil and groundwater or downstream of a point-source discharge. Additional studies on the human and ecological risk of pharmaceuticals at environmentally relevant levels, including synergistic effects with other chemicals, would be beneficial for an accurate exposure assessment.

Pharmaceuticals represent a class of several hundred chemicals that together with their human and environmental metabolites present a major challenge in terms of developing standardized, reliable sampling and analytical methods with adequate quality control. Such methods could be built around a prioritized list that takes into account production and use, their predicted “impact” based on toxicity and activity, and their degradation and persistence in WWTP effluents; however, a critical data review process should be in place to ensure methodological credibility and to provide a sound basis for source characterization and exposure assessment.

The bioconcentration of pharmaceuticals in indicator species, such as aquatic life and their predators, could provide a better measure of environmental loading and accumulation because they are closer to sources of pollutants.

Pharmaceutical agents and their human metabolites are being discharged into the aquatic environment at a loading that is related to the size and the age of people in the community and the dynamic relationship between WWTP discharge and environmental exposure. This loading is likely to be affected by three major factors: *a*) the production of a more concentrated effluent from WWTPs due to the increase in population density and the number of elderly people in the community, *b*) the reduced dilution of sewage as a consequence of sustained drought conditions that results in a reduced flow of receiving streams and an increased proportion of treated wastewater reaching downstream reservoirs, and *c*) an increase in the number of communities moving to the use of reclaimed water because of water scarcity and climate change.

Sources of drinking water (both surface and ground) are increasingly being affected by upstream WWTP discharges or soil–aquifer infiltration, but it remains to be demonstrated whether the technology used in processing wastewater for reclamation is selective in removing pharmaceutically active chemicals or whether they can be concentrated through sediment transport. Evidence to date suggests that the simplest and most commonly used conventional WWTPs are only partially effective in removing the most hydrophobic components and, in so doing, concentrate a wide range of organics, including pharmaceuticals (Chenxi et al. 2008), in biosolids that are often sold as fertilizer, which when irrigated could release the chemicals into the ground.

### Exposure effects and risks to humans and ecosystems

When assessing environmental and health risks from exposure to chemical pollutants, it is important to clearly distinguish between humans and ecosystems in terms of both exposure and effects. The effects of parts per billion or lower concentrations on ecosystems can range from changes in gene expression to changes in population structure, although little evidence exists for such adverse effects from most pharmaceuticals. However, two extraordinary examples do exist. The best known was the dramatic decrease in vulture populations in India and Pakistan (95% in 3 years), where vultures that fed on carcasses of cattle treated with diclofenac died from renal failure because they were unable to excrete the drug (Oaks et al. 2004). The other case involved the deliberate dosing of an entire experimental lake with low levels of the active ingredient in birth control pills, ethinyl estradiol. Within the first year, fathead minnows showed evidence of responses at the cellular and tissue levels and declines in the population; by the second year, the fish population had collapsed completely (Kidd et al. 2007). Other studies have shown evidence of endocrine disruption effects in fish populations (e.g., intersex, histological changes in gonads, feminization of male fish preventing them from reproducing) exposed to effluents that contain chemicals such as ethinyl estradiol (Jobling et al. 1998); the predicted no-effect concentration for aquatic effects from this compound is < 1 ppt (Caldwell et al. 2008), but levels of this chemical have been found at up to 27.4 ppt in the river waters of Taiwan (Chen et al. 2007) and at up to 178 ppt in WWTP effluents in Australia (Fernandez et al. 2007).

In aquatic ecosystems, the fact that many of these chemicals are designed to resist biodegradation favors their adsorption onto watershed sediments, so despite the low aqueous levels reported, we do not yet have a grasp of the implications of their constant infusion into rivers and streams. Could this, for example, be generating a hidden environmental risk associated with long-term exposure and combinatory effects? The case of antibiotics could be enlightening; for example, a study in North Carolina (USA) has suggested that a defined environmental effect, the development of antibiotic resistance, may be evolving in the aquatic–sediment interface where multiple antibiotics are accumulating (Stauffenberg and Weinberg 2006).

The primary exposure pathways to humans other than those from prescribed dosing are through drinking water at part-per-trillion levels (Stackelberg et al. 2007; Ye et al. 2007), which for typical daily consumption over a lifetime, would provide exposure to individual compounds well below a single therapeutic dose and suggest little threat to human health (Fent et al. 2006), although the effects on pregnant women and their fetuses are still not clear. Exposure through bathing in contaminated recreational waters, the potential for pharmaceuticals to migrate across the skin barrier, and contact with antibiotic-resistant bacteria are all potential secondary exposure pathways and examples of research needs for assessing the impact of pharmaceuticals in the environment (Ankley et al. 2007).

Approaches to the evaluation of toxicity associated with chronic low-dose exposure to mixtures of pharmaceuticals vary depending on whether we consider human or ecological hazard risk assessment. As an example, surveillance of waters receiving wastewater effluents is critical for ecological hazard risk assessment, whereas biomonitoring or chemical analysis of drinking water is more important for human hazard risk assessment. However, it is critical to ensure that the correct end points are being considered and that they are plausible to measure in low-dose chronic exposures. There is, therefore, a need to consider nontraditional end points of toxicity, such as behavioral, developmental, and reproductive responses, including toxicogenomics, relevant “omics” techniques in general, or others such as “no observable transcriptional effect levels.” A need also exists to identify organisms or subsets of populations, including specific life stages (e.g., fetal development), that might be more vulnerable to pharmaceuticals. In particular, assessment of pharmaceuticals must consider the mixture of compounds with which exposure occurs. The assumption that additive models do not adequately assess effects of pharmaceuticals has not been rigorously tested, so the applicability of existing screening methods must be evaluated. Alternate ways to address or evaluate mixtures can be considered, including a screening-threshold approach where the threshold of toxicologic concern is the denominator (Daughton 2008; Kroes et al. 2005), the measured or predicted exposure concentration is the numerator, and the resulting ratio is evaluated: If < 1, no further actions are warranted. Alternatively, an integrated epidemiological, biomonitoring, and demographic approach could be used. In fact, such an approach was recently applied to the assessment of a different category of drinking water contaminants, namely, mixtures of disinfection by-products that were formed during the treatment of drinking water (Simmons et al. 2008). That study could serve as a model to frame the evaluation of mixtures of pharmaceuticals.

Risk assessment could also benefit from a prioritization listing of pharmaceuticals to help determine which pose the highest risk based on specific factors such as mode of action, therapeutic dose, and environmental exposure. Kostich and Lazorchak (2008) recently published a model to help implement this pharmaceutical prioritization approach, but no widely accepted prioritization list yet exists. It would also be prudent to test whether the existing human health data on pharmaceuticals can be applied to environmental hazard risk assessment even when the environmental doses are much lower than therapeutic doses. Ecosystem adaptation and the changes in sensitivity of organisms (increased or decreased) with continuous exposure are other critical issues to be considered in future health and ecological risk studies.

### Best management practices

Despite the various complex variables that combine to cause pollution of the aquatic environment by pharmaceuticals, actions can be taken to reduce their presence in the environment, such as responsible disposal of leftover drugs from the consumer sector. Some activities have already been sponsored by the U.S. federal government regarding drug disposal (Daughton 2003). As an example, the White House Office of National Drug Control Policy, in collaboration with the U.S. EPA and FDA, implemented the nation’s first public guidance on consumer drug disposal (Office of National Drug Control Policy 2009), but guideline standardization is still needed.

The most effective best management practices (BMPs) would address source reduction, namely, reducing the amount of medicine that goes unused. The Pharmaceutical Research and Manufacturers of America have evaluated unused medicine disposal options and have concluded that toilet flushing of unused medicine should be avoided whereas household trash disposal and take-back programs are effective at removing the unused medicine contribution to pharmaceuticals in water (SMARXT DISPOSAL 2009). Another approach for a more environmentally sound handling of unused drugs is to use “social marketing” (Newton-Ward 2007). Marketing technologies would be applied to the analysis, planning, execution, and evaluation of programs designed to influence the voluntary behavior of target audiences toward drug disposal.

Before implementation of BMPs can be assured and widely practiced, the following questions need to be addressed:

- What are the roles and responsibilities of players within the various interconnected domains, namely, the industrial, medical, social, and environmental sectors?
- Which metrics should be used to design and assess BMPs; are they currently being deployed, and how predictive are they?
- What achievable BMPs can be established for contaminants already known to cause adverse effects?
- What can be done immediately with what we know today, and which BMPs are practical and cost-effective to implement?

Responses to these questions will differ depending on the sector considered. Supplemental Material, Table 1 (available online at doi:10.1289/ehp.0901532) suggests the most feasible BMPs for short-term implementation, which are presented in order of priority according to such criteria as speed of implementation, cost containment, and efficacy.

The production, transport, and fate of pharmaceuticals cross the jurisdiction of multiple regulatory agencies in the United States, creating a challenge to balance the societal need for safe and effective drugs against possible deleterious human and environmental impacts from the presence of their residues in the environment. Better interagency cooperation, data sharing, a focal point for communication, and overall better federal facilitation are essential to seriously address public concerns on these issues, which include the judicious evaluation and effective solving of any questions or problems that arise. During summit discussions, a consensus was reached that some organization (which, in the United States, could be the U.S. EPA or another federal agency) needs to oversee the specific issue of pharmaceuticals in drinking water to ensure justification for action or lack thereof. The U.S. Congress might designate leadership to provide guidance and background on existing efforts, but stakeholders have to be involved in the evolution of process, and these should include at a minimum with pharmaceutical companies, water utilities, regulatory agencies, and academia.

### Education and communication

Three key areas were suggested for education and communication: transparency, research into communication, and focus of education.

#### Transparency

The implications of the presence of pharmaceuticals in water should be communicated to the public in a truthful manner without raising alarm or suspicion and causing mistrust.

#### Research into communication

The target audience has to be identified, as well as their perception of the subject before communiqués are distributed. Potential audiences would include the general public, legislators, farmers, regulators, health and food industry representatives, water treatment plant and WWTP operators, journalists, and scientists.

#### Focus of education

Those who prescribe and dispense medications, farmers, and the general public should be educated with the objective of creating interventions with the most likely and immediate impact.

Supplemental Material, Table 2 (doi:10. 1289/ehp.0901532) provides initial recommendations for better communication and education, prioritized in terms of feasibility for short-term implementation.

One specific goal established during the summit was to come up with a simple and transparent response to public and media concerns about the safety of the drinking water supply related to pharmaceutical contamination. In response to this task, the summit participants issued the following statement:

“If your drinking water meets current U.S. standards, your drinking water is considered safe and drinkable. We recognize that trace amounts of pharmaceuticals in combination with other chemicals have been found in water. These substances are coming from a variety of sources and are difficult to completely remove. There is limited information on how long-term, low-dose exposures affect humans and wildlife. U.S. standards may need to be developed for pharmaceutical compounds in drinking water or in aquatic systems as more information becomes available.” (Research Triangle Environmental Health Collaborative 2008).

Although not wishing to convey a false sense of security to the public by declaring the water supply risk-free from the effects of pharmaceuticals, it is important to stress that risk assessment is ongoing. Moreover, even if it turns out that pharmaceuticals are not a major concern, there are still other chemicals (e.g., personal care products, pesticides, industrial contaminants, etc.) that could pose a human health or ecological risk.

## Conclusions and Recommendations

Despite rising fears over the presence of pharmaceuticals in drinking water, there is currently little evidence associating them with adverse human health risks, but this summit served to highlight the issues of concern that need to be addressed to confirm this perception. Supplemental Material, Table 3 (doi:10.1289/ehp.0901532) collates the major research needs in this respect. For example, more and better data are needed to prioritize which pharmaceutical chemicals could potentially pose the highest risk to consumers and to the environment. The identification of those drugs susceptible to formation of degradation and/or transformation products that have equal or more toxicity than the parent compounds is a critical point in this respect. Effectiveness of current wastewater treatment, on-site processes, and emerging animal waste treatment technologies also needs to be evaluated.

In general, ecosystems appear to be more at risk than humans in part because aquatic organisms may have a higher sensitivity to and may be exposed to higher levels of pharmaceutical residues than are humans. The key challenge in assessing the risk of pharmaceuticals in the environment is to evaluate whether unintended long-term, low-dose exposures to mixtures of these chemicals can potentially affect humans and wildlife. In this respect, questions regarding susceptible subpopulations or life-stage sensitivity (e.g., the fetus) must be considered. Existing frameworks, such as those developed for pesticides and disinfection by-products, including the use of tracers or surrogates for whole groups of pollutants, may be appropriate for use as models assessing hazard or risk of pharmaceuticals in the environment.

BMPs have to be implemented covering the industrial, medical and veterinary, social and agricultural, and environmental spheres. Most BMPs suggested in this review focus on education of the health care community and general public on the proper disposal of unused medicine and how steps can be taken to reduce the amount of medicine that goes unused. Other BMPs listed include take-back programs, evaluation of treatment technologies, and increased monitoring of pharmaceuticals in water. An evaluation of the effectiveness of BMPs once they have been implemented would be desirable, but there needs to be supervision or oversight for coordination of all activities related to studying the specific issue of pharmaceuticals in drinking water and their sources.

Even though there is limited information about environmental and public health effects derived from the presence of pharmaceuticals in water, there is a need to communicate what is known. The use of new technologies is recommended for facilitating communication and collaboration. However, the information must be clear, without providing mixed messages on the subject of potential yet currently unknown risks, remaining truthful without causing alarm. If the idea that water can be considered safe is communicated to the public, it must be emphasized that this is based on the test data of scientific studies, lack of observed adverse effects where these have been evaluated, and current regulations.

Water scarcity, climate change, aging and increasing population density, increasing use of pharmaceutical products, and rising dependence on water reuse may lead to an increase in the presence of pharmaceuticals in groundwater, surface water, and drinking water in the near future that might pose a risk to water safety or an exacerbation of perceived risk. Scientific techniques to understand and predict the potential for long-term effects of pharmaceutical residues in the environment are continuing to be developed to assess these challenges and to help prevent environmental and human health effects.

The summit provided an interdisciplinary forum that considered a multipronged approach to a potential public health challenge. It demonstrated the benefit of collecting voices from those who would normally work in disconnected environments and provides a “clearinghouse” or umbrella through which congressional offices and other interested parties can seek information on the subject discussed. More such interactions among scientists, public advocacy groups, and representatives from relevant industries, other stakeholders, and policy makers will help to clearly define the challenges faced by special issues and to provide each sector with a better understanding on how best to assimilate and disseminate their knowledge. In turn, this will help scientists to better advocate for public policies.
